# CCR7 mediates human breast cancer cell invasion, migration by inducing epithelial–mesenchymal transition and suppressing apoptosis through AKT pathway

**DOI:** 10.1002/cam4.1039

**Published:** 2017-04-04

**Authors:** Bing Xu, Minjie Zhou, Wencai Qiu, Jueming Ye, Qiming Feng

**Affiliations:** ^1^ Department of Emergency MedicineShanghai Jiao Tong University Affiliated Sixth People's HospitalShanghai200233China; ^2^ Department of General SurgeryShanghai Jiao Tong University Affiliated Sixth People's HospitalShanghai200233China

**Keywords:** Apoptosis, breast cancer, CCR7, epithelial–mesenchymal transition, invasion

## Abstract

Chemokine and the chemokine receptor have a key role in the tumor progress. Here, we supposed that CCR7 might induce the invasion, migration, and epithelial–mesenchymal transition (EMT) process of breast cancer. In this research, human breast cancer MCF‐7 and MDA‐MB‐231cells were treated with CCL19 and small‐interfering RNA (CCR7 siRNA) for activation and inhibition of CCR7, respectively. Cell invasion and transwell assays were used to detect the effect of CCR7 on invasion and migration. The results demonstrated that CCL19 mediated cell invasion and migration by inducing the EMT, with downregulation of E‐cadherin and up‐regulation of N‐cadherin and vimentin levels. On the other hand, knockdown of CCR7 revealed the changes compared with CCL19 group and the control group. Knockdown of CCR7 inhibits CCL19‐induced breast cancer cell proliferation, the cell cycle, migration, invasion and EMT. Moreover, we demonstrated that CCL19‐induced AKT phosphorylation; however, CCR7 siRNA suppressed CCL19‐induced AKT phosphorylation, a key regulator of tumor metastasis. In conclusion, all findings demonstrated that CCL19/CCR7 axis regulated EMT progress in breast cancer cells and mediated the tumor cell invasion and migration process via activation of AKT signal pathway. Our results suggested that CCR7 may regard as a therapeutic target for the breast cancer treatment.

## Introduction

Breast cancer is the most common diagnosed tumor and the second main cause of cancer mortality in women worldwide 1, 2. About 60–70% distant metastases happened in the breast cancer patients 3, 4. Tumor metastasis is a key risk factor for the survival of breast cancer patients and other cancer 5, 6. Metastasis is a complicated progression involving in cell proliferation, migration, and invasion 7. Therefore, understanding the molecular mechanisms of breast cancer progression and metastasis would reveal effective diagnostic targeted therapy.

Recently, the epithelial–mesenchymal transition (EMT) has regarded as a key progress in cancers development 8, 9, 10. With the EMT progress development, epithelial cells reduced their cell polarity and cell–cell adhesion, and mesenchymal cells attained migration and invasion ability. After EMT progress, epithelial cancer cells decreased E‐cadherin expression and induced N‐cadherin, fibronectin, and vimentin levels. Moreover, EMT progress triggered the transcription factors Snail and Twist expression that suppressed E‐cadherin expression 11. Therefore, knowing the molecular mechanisms of EMT progress is crucial to the treatment of breast cancer.

In the tumor microenvironment, chemokines and chemokine receptors have essential roles in tumor proliferation, metastasis, and invasion. CC‐chemokine receptor 7 (CCR7) was confirmed to participate in cancer cell metastasis 12, invasion 13, and tumor development 14. In the experimental breast cancer model, CCR7 had a novel role in the stimulation of lymphangiogenesis 15. Furthermore, CCR7 was accompanied with EMT in human breast carcinoma 16 and gastric cancer 12. Moreover, CCR7 was involved in the lymph node metastasis, invasion, migration, and EMT have previously been reported 12. In clinical studies, the high expression of CCR7 is related with poorer prognosis and shorter survival rate 17, 18, suggesting that CCR7 was involved in the development and recurrence of breast cancer. However, its role in breast cancer and the molecular mechanisms remain elusive.

In the present study, we found that CCR7 knockdown inhibited CCL19‐induced cells motility and invasion. Moreover, we also observed that CCR7 silencing altered CCL19 promoted EMT. CCR7 promoted EMT and apoptosis via AKT pathway, which indicated that CCR7 has a key role in breast cancer development progression. Thus, the CCL19–CCR7 axis may provide potential targeting molecules for advanced breast cancer therapy.

## Materials and Methods

### Cell culture and CCR7 ‐siRNA transient transfection

Human breast cancer MCF‐7 and MDA‐MB‐231 cell lines were purchased from the Cell Bank of Chinese Academy of Sciences (Shanghai, China). MDA‐MB‐231 and MCF‐7 cells were cultured in Dulbecco's modified Eagle's medium (DMEM) (Gibco, Carlsbad, CA) with 10% fetal bovine serum (FBS) (Hyclone, Logan, UT) at 37°C in a humidified atmosphere of 5% CO_2_.

Cells were transfected with either CCR7 siRNA by lipofectamin 2000 (Invitrogen, Carlsbad, CA) according to the manufacturer's instructions. Cells were harvested at 72 h following transfection of CCR7 siRNA (CCR7 gene: 5′–GAAGUGCAUACACCGAGAC–3′). Efficiencies of CCR7 siRNA were detected by qPCR and western blot.

### Migration and invasion assay

Transwell inserts (24‐mm diameter, 8‐*μ*m pores, Costar, Corning, NY) were used to detect cells migration and invasion. For invasion assay, each insert was pre‐coated with Matrigel (BD Biosciences, Becton, CA). Cells (2 × 10^5^) were cultured in DMEM with 0.5% FBS and added in the upper chamber, whereas DMEM with 5  ng/mL CCL19 (R&D Systems, Inc., Minneapolis, MN, USA) was seeded to the lower chamber. After incubation at 37°C for 24 h, the noninvasive cells were removed. Invasive cells were fixed in 4% paraformaldehyde and stained with hematoxylin. The invasive cells were calculated under light microscopy (×100 magnification). In the migration assay, the upper inserts were without the Matrigel precoating.

### Cell proliferation assay

The cells (1 × 10^5^) were seeded into 96‐well plates. At the indicated times, the cells were incubated with 1 mg/mL MTT in DMEM for 6 h at 37°C. The formazan was dissolved in 200 *μ*L dimethyl sulfoxide. The optical density (OD) of the formazan solution was measured at 570 nm by a microplate reader and the experiments were repeated three times.

### Cell cycle analysis

The cells (1 × 10^6^) were stained with 50 *μ*g/mL propidium iodide (Sigma‐Aldrich, St. Louis, MO, USA) and 1 mg/mL RNase A (Invitrogen) for 30 min at 37°C in the dark. Samples of the cells were then analyzed for their DNA content using flow cytometry, the data were analyzed by BD FACSDiva^™^ Software (BD Biosciences, Franklin Lanes, NJ). All experiments were performed in duplicate and repeated three times.

### Measurement of caspase‐3 activity

The caspase‐3 activity was detected at a wavelength of 405/650 nm (excitation/emission) using a caspase‐3 colorimetric assay kit according to the manufacturer's protocol.

### Matrix metalloproteins (MMPs) activity assay

The MMP‐9 and MMP‐2 activity were revealed by QuickZyme MMPs activity assay (QucikZyme BioSciences) according to the manufacturer's protocol. The cells supernatant were added to the 96‐well plates coated with MMP‐9 or MMP‐2 antibody at 4°C overnight. Then, 50 *μ*L assay buffer and detection reagent were added at 37°C for 1 h. The OD_405_ was measured by a microplate reader.

### Real time quantitative PCR analysis (qPCR)

The total RNA was extracted from the cells using TRIzol reagent (Invitrogen, Life Technologies) for reverse transcription. The cDNAs were synthesized with Transcriptor Reverse Transcriptase (Roche , Basel, Switzerland) according to the manufacturer's protocol. RT‐PCR was performed following the procedures described previously. The CCR7 primers: sense, 5′–CCCTTGGGTGTCAAAGGTAAA–3′ and antisense, 5′–AAACTGATGCGTGAAGTGCTG–3′; and *β*‐actin sense, 5′–GCGAGCACAGAGCCTCGCCTTTG–3′ and antisense, 5′–GATGCCGTGCTCGATGGGGTAC–3′.

### Western blot

Total proteins were extracted in RIPA Lysis buffer (Millipore, Temecula, CA) and calculated by the BCA method. The protein were resolved by 10% sodium dodecyl sulfate (SDS)‐PAGE and transferred to nitrocellulose membrane (Millipore). The membrane was incubated overnight with the primary antibodies and horseradish peroxidase‐conjugated secondary antibodies for 1 h. The immunoblots were detected by an ECL Kit (Millipore), according to the manufacturer's protocol.

### Akt1 ‐siRNA transient transfection

Akt1 siRNA and control siRNA were purchased from Cell Signaling Technology. MCF‐7 cells were transfected with siRNA (100 nmol/L) according to the manufacturer's protocol. Cells were harvested at 48 h, and the migration and invasion assays were performed. Efficiencies of Akt1 siRNA were detected by western blot.

### Statistical analysis

Data are expressed as the mean ± SD. The statistically significant differences between untreated control and drug‐treated cells were evaluated using Student's one tailed *t* test (in all figures, *indicates *P* < 0.05, whereas ** indicates *P* < 0.001).

## Results

### CCL19 increases the invasion, migration, and EMT of breast cancer cells

**Figure 1:**
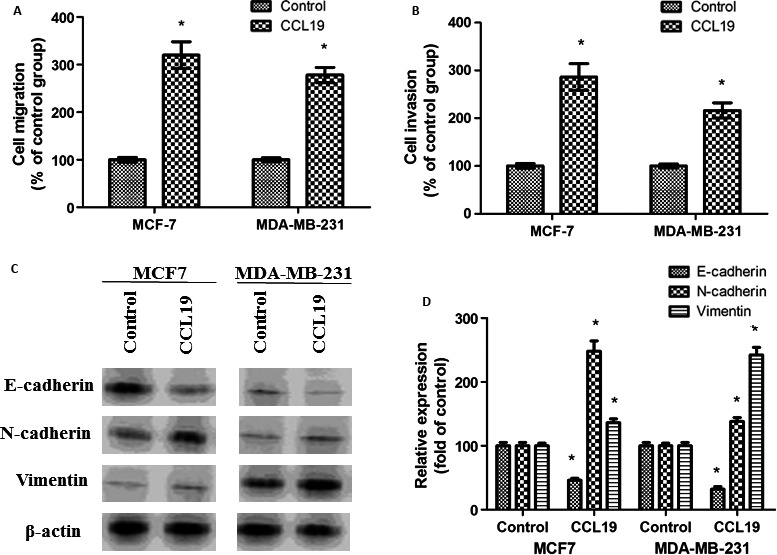
CCL19 induces breast cancer cells invasion, migration, and EMT in vitro. (A) Cells were detected for the migration ability after stimulation with CCL19. (B) The invasion ability of CCL19‐treated cells significantly increased. (C) CCL19‐induced breast cancer cells EMT progress. (D) Quantitative western blot analysis of EMT expression. Data are expressed as mean ± SD from three independent experiments. *P <* *0.05 (as compared with control cells).

First, we detected whether CCL19 could promote breast cancer cell progression. CCL19 enhanced cell migration (Fig. 1A) and invasion (Fig. 1B) of breast cancer cells.

Moreover, CCL19 decreased the epithelial marker level, E‐cadherin expression, whereas increased the mesenchymal marker level, N‐ and vimentin expression. (Fig. 1C and D).

### Knockdown of CCR7 inhibits CCL19‐induced migration and invasion

**Figure 2:**
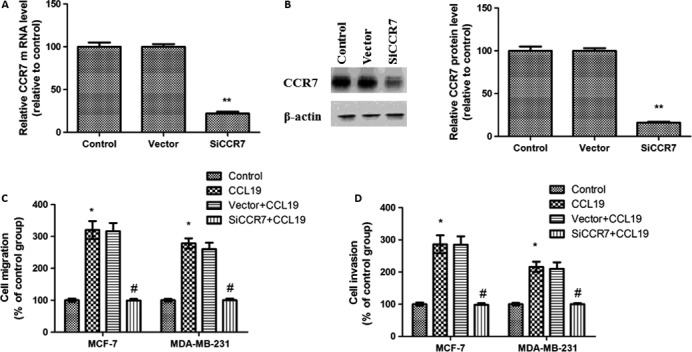
Knockdown of CCR7 decreased breast cancer cells migration and invasion in vitro. (A) Quantitative polymerase chain reaction analysis of CCR7 after RNAi silencing. (B) Western blot analysis of CCR7 expression. (C) CCR7 inhibition on the CCL19‐induced migration of breast cancer cells. (D) CCR7 inhibition significantly affected the CCL19‐induced invasion of breast cancer cells. Data are expressed as mean ± SD from three independent experiments. *P <* *0.05 (as compared with control group), ^#^ *P *<* *0.05(as compared with CCL19 group).

To study the responsibility of CCR7 in CCL19‐induced tumor progression, CCR7 were inhibited by SiRNA. As shown in Figure 2A and B, CCR7 mRNA and protein expression levels were obviously reduced.

Next, we also detected the role of CCR7 in CCL19‐induced tumor migration and invasion. CCL19‐induced cell migration was abolished in CCR7 siRNA cells (Fig. 2C). Furthermore, CCL19‐induced cell invasion was reduced in CCR7 siRNA cells (Fig. 2D).

### CCR7 siRNA affected the expression of EMT biomarkers

**Figure 3:**
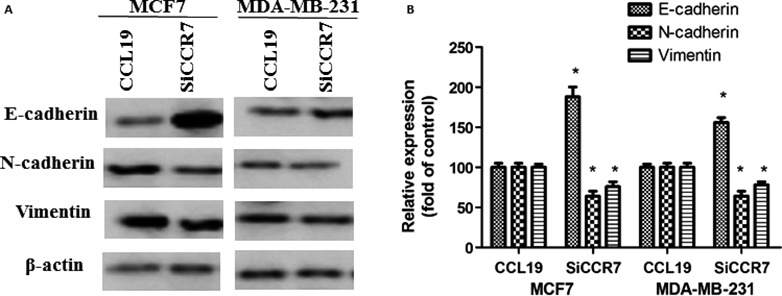
CCR7 inhibition significantly affected the CCL19‐induced EMT progress of breast cancer cells. (A) Western blot analysis to determine the levels of EMT protein. (B) Quantitative analysis of the protein expression as shown in A. Each bar represents mean ± SD from three independent experiments. *P < 0.05 (as compared with control group), #P < 0.05(as compared with CCL19 group).

To confirm our hypothesis, we evaluated the EMT biomarkers levels including vimentin, N‐cadherin, and E‐cadherin. In this study, the data showed CCL19‐induced vimentin and N‐cadherin levels, moreover, reduced E‐cadherin level. In contrast, CCR7 siRNA notably regulated EMT biomarker expression in comparison with CCL19‐treated group (Fig. 3).

### CCL19 regulates breast cancer cells’ cell proliferation and the cell cycle

**Figure 4:**
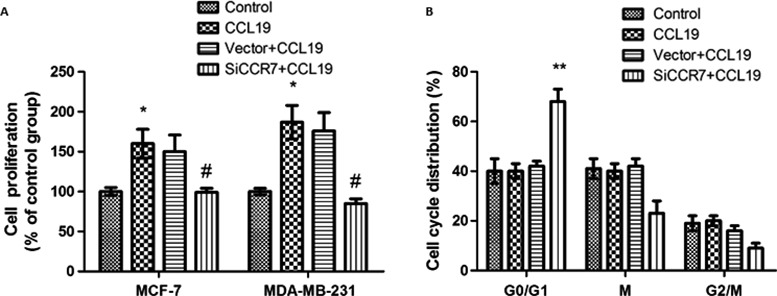
CCR7 inhibition significantly affected the CCL19‐induced cell proliferation and the cell cycle in breast cancer cells. (A) SiCCR7 affects CCL19‐induced cell proliferation. (B) SiCCR7 affects cell cycle in breast cancer cells. Data are expressed as mean ± SD from three independent experiments. *P <* *0.05 (as compared with control group), ^#^ *P *<* *0.05 (as compared with CCL19 group).

To evaluate the effect of CCL19 in breast cancer cells proliferation and the cell cycle, cell proliferation and the cell cycle were measured by MTT assay and flow cytometry assay. These results indicated the proliferation of cells was increased compared with the control group (Fig. 4A). However, CCL19 had no effect on cell cycle arrest in G0/G1 phase in cells (Fig. 4B).

### Knockdown of CCR7 inhibits CCL19‐induced breast cancer cell proliferation and the cell cycle

To measure the influence of CCR7 siRNA on CCL19‐induced breast cancer cells proliferation and the cell cycle, we also examined by MTT assay and flow cytometry assay. After CCR7 siRNA treatment, the proliferation of cells induced by CCL19 was reduced (Fig. 4A).

In CCR7 siRNA group, the number of cells in G0/G1 phase was enhanced compared with control group. These results indicated that CCR7 silencing can induce cell cycle arrest in G0/G1 phase in cells (Fig. 4B).

### CCR7 siRNA reduced AKT signaling pathway and induced caspase‐3 and MMPs activity

**Figure 5:**
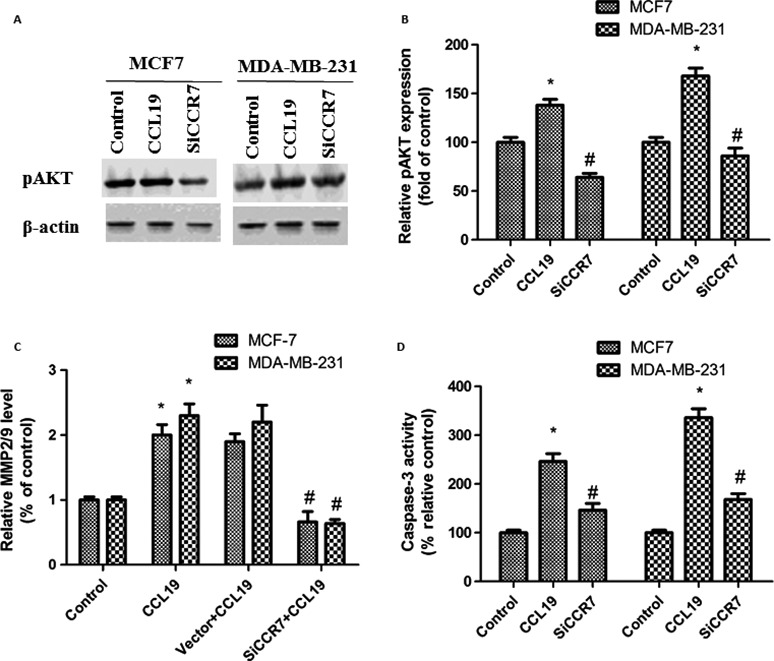
CCR7 inhibition significantly affected the CCL19‐induced AKT signaling pathway and caspase‐3, MMPs activity. (A) SiCCR7 affects pAKT expression by western blot. (B) Western blot analysis of pAKT expression. (C) SiCCR7 affects MMP2/9 activity expression by ELISA. (D) SiCCR7 affects caspase‐3 activity expression. Data are expressed as mean ± SD from three independent experiments. *P <* *0.05 (as compared with control group), ^#^ *P *<* *0.05 (as compared with CCL19 group).

CCL19 treatment upregulated p‐AKT expression, implying that AKT pathway was activated. To measure the influence of CCR7 siRNA on AKT pathway in breast cancer cells, we detected the p‐AKT expression by western blot. After CCR7 siRNA treatment, the p‐AKT expression was decreased (Fig. 5A and B). All results confirmed that AKT pathway was fundamental for the potential roles of CCR7.

In order to study the mechanisms of the decreased invasion and migration ability by CCR7 siRNA treatment, MMP‐2/9 expression in cells were measured by ELISA. As shown in Fig. 5C, the MMP‐2/9 levels in the CCR7 siRNA groups were reduced compared with control groups.

In order to study the mechanisms of the increased apoptosis ability by CCR7 siRNA treatment, caspase‐3expression in cells were measured by a colorimetric assay kit. As shown in Fig. 5D, the caspase‐3 levels in the CCR7 siRNA groups were enhanced compared with control groups.

### Inhibition of AKT signaling reverses CCL19‐induced MCF‐7 cells EMT progress

**Figure 6:**
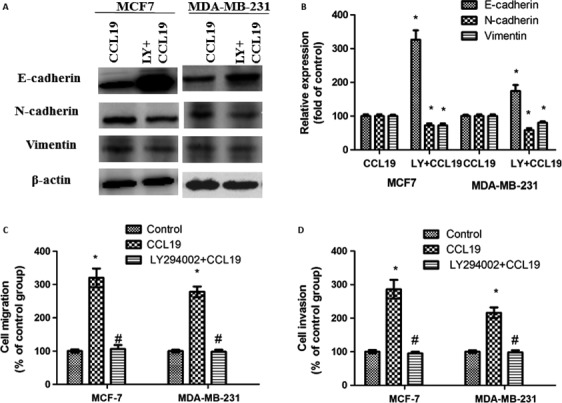
Suppression of AKT signaling pathway reverses CCL19‐induced MCF‐7 cells EMT progress, migration, and invasion. (A) SiCCR7 affects CCL19‐induced cell EMT. (B, C) SiCCR7 affects CCL19‐induced cell migration. (D) SiCCR7 affects CCL19‐induced cell invasion. All data are presented as mean  ±  SD from three independent experiments. *P <* *0.05 (as compared with control group), ^#^ *P *<* *0.05(as compared with CCL19 group).

We have previously indicated that CCL19 (5  ng/mL) enhanced EMT in MCF‐7 cells. To detect the AKT pathway involved in CCL19‐induced EMT progress, we used the chemical inhibitor LY294002. After treatment with the LY294002, the expression of vimentin and N‐cadherin decreased, and the expression of E‐cadherin increased, suggesting that inhibitor LY294002 reverses CCL19‐induced EMT progress (Fig. 6A and B).

### Suppression of the AKT pathway reduces CCL19‐induced MCF‐7 cells potential of motility and invasion

The capacity of breast cancer cells regulated EMT progress exhibited an enhanced metastatic and invasion abilities. We next detected the AKT pathway involved in CCL19‐induced MCF‐7 cells migration and invasion. After treatment with the LY294002, both the migration (Fig. 6C) and invasion (Fig. 6D) reduced, suggesting that inhibition of the AKT pathway reverses CCL19‐induced migration and invasion ability.

### Inhibition of AKT signaling by Akt1 siRNA reverses CCL19‐induced migration, invasion, and the secretion of MMP‐2/9 in MCF‐7 cells

**Figure 7:**
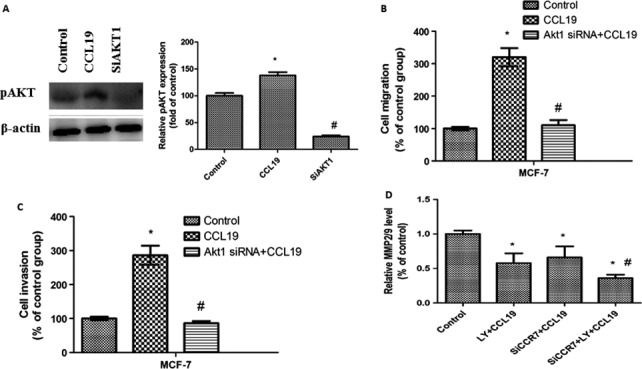
Suppression of AKT signaling pathway reverses CCL19‐induced MCF‐7 cells migration, invasion, and the secretion of MMP‐2/9. (A) SiAKT1 affects pAKT expression by western blot. (B) SiAKT1 affects CCL19‐induced cell migration. (C) Si AKT1 affects CCL19‐induced cell invasion. All data are presented as mean  ±  SD from three independent experiments. *P <* *0.05 (as compared with control group), ^#^ *P *<* *0.05(as compared with CCL19 group). (D) MCF‐7 cells transfected with SiCCR7 were treated with or without LY294002, and the MMP2/9 activity expression by ELISA. All data are presented as mean  ±  SD from three independent experiments. *P <* *0.05 (as compared with control group), ^#^ *P *<* *0.05(as compared with the CCR7 silencing or treatment with the inhibitor alone group).

To measure the influence of AKT pathway in CCL19‐meditated breast cancer cells, we also used Akt1 siRNA. After Akt1 siRNA treatment, the p‐AKT expression was decreased (Fig. 7A). We next detected the AKT pathway involved in CCL19‐induced MCF‐7 cells migration and invasion. After treatment with the Akt1 siRNA, both the migration (Fig. 7B) and invasion (Fig. 7C) reduced, suggesting that inhibition of the AKT pathway reverses CCL19‐induced migration and invasion ability. The results were similar to that reported by LY294002 (Fig. 6), the data implied CCL19‐induced migration and invasion of MCF‐7 cells via AKT signal pathway.

Finally, the expression of MMP‐2/9 in the siCCR7 treatment of MCF‐7 cell that had been pre‐treated with LY294002 were further reduced, as compared with that in the LY294002‐treated and siCCR7‐treated cells (Fig. 7D). Altogether, our data suggested that either suppression of the AKT signal or knockdown of CCR7 reduced the secretion of MMP‐2/9, and that both suppression of the AKT signal and CCR7 silencing synergistically decreased the secretion of MMP‐2/9 in MCF‐7 cells.

## Discussion

Several past researches indicated high level of CCR7 associates with tumor metastases and poor clinical outcome in many kinds of cancer, including esophageal cancer 19, lung cancer 14, and other cancer 12, 20, 21, 22. Some other studies have reported that CCR7 mediates chemotactic process, such as promotion of angiogenesis and lymphangiogenesis 23. Nevertheless, whether CCR7 is involved in the EMT progress of human breast cancer is unknown. The aims of our study were to explore the effect of CCR7 on the EMT of breast cancer cells and the underlying mechanisms. Our data exhibited that knock‐down of CCR7 reduced the EMT, migration, and invasion of breast cancer cells. Furthermore, knock‐down of CCR7 inhibited phosphorylation of AKT expression. In addition, CCR7‐mediated phosphorylation of AKT expression and cell EMT, migration, and invasion significantly reduced after treated with LY294002. Taken all together, our findings implicated the involvement of CCR7 in EMT, migration, and invasion of breast cancer cells, which may be through AKT signaling pathway.

In the present study, we confirmed that CCL19 induces the invasion and migration of breast cancer cells. We also found that CCL19 induces the expression of vimentin and N‐cadherin, and reduces the expression of E‐cadherin. At the same time, we used siRNAs to detect the effects of CCR7 in vitro. Knockdown of CCR7 inhibits CCL19‐induced migration and invasion. Moreover, blockade of CCR7 notably regulated EMT biomarker expression. All these results implied that CCR7 was indeed implicated in the EMT process. EMT progress is triggered by various growth factors, and is regulated by signal networks, including PI3K/AKT signal, which has been shown to block E‐cadherin and enhance snail transcription expression 24, 25, 26.

After EMT progress development, many kinds of cancer cells enhanced migration and invasion abilities 27, 28, 29. The major alteration that occurs during EMT progress is the continuous decreased E‐cadherin level and the increased N‐cadherin expression 30. This E‐cadherin/N‐cadherin switch is triggered by many transcription factors, such as Slug, Snail, and Twist; while all these transcription factors suppress the expression of E‐cadherin, Twist stimulates the expression of N‐cadherin 31. The earlier research has indicated that Slug, Snail, and Twist maybe regulated by the PI3K/AKT pathway 32, 33, 34. As expected, AKT phosphorylation induced by CCL19 was also repressed by siRNA CCR7. AKT pathway provoked cell survival and may induce cell migration and invasion. It was reported that CCL19/CCR7 responded for the migration of cancer calls via the AKT pathway 24. Our evidence identified AKT as being associated with the EMT process, indicating CCR7 was implicated in EMT progress development via AKT pathway.

Moreover, anti‐activation of the PI3K/AKT pathway in CCR7‐knockdown breast cancer cells causes of decreased N‐cadherin expression. We therefore hypothesized that the suppression of E‐cadherin/N‐cadherin switch that occur in the CCR7‐knockdown cells during EMT progress is a direct result of the inhibition of PI3K/AKT signal. So, CCR7 may be the key factors that elevate the EMT process in breast cancer.

MMPs had a key role in the invasion and migration of tumor cells. We found that knockdown of CCR7, similar to that suppression of the AKT signal pathway, markedly decreased the secretion of MMP‐2/9 in MCF‐7 cells. These results implied that the AKT pathway is essential for the MMP secretion in MCF‐7 cells. It is possible that CCR7 trigger the AKT activation, eventually leading to MMP‐2/9 secretion. Therefore, inhibiting CCR7 is a therapeutic targeting for suppression the AKT activation, MMP‐2/9 expression, and attenuating the migration, invasion and EMT of MCF‐7 cells.

Taken all together, our results demonstrated that CCR7 participated in various processes in breast cancer progress. Our study suggested that CCR7 mediates EMT progress via AKT pathway, which indicated that CCR7 has a key role in breast cancer progression. Thus, our studies elucidating the CCR7 could be a novel target for tumor therapy.

## Conflict of Interest

The authors did not report any conflict of interest.

## Ethical Approval

This article does not contain any studies with animals performed by any of the authors.
